# Improving Accuracy of Heart Failure Detection Using Data Refinement

**DOI:** 10.3390/e22050520

**Published:** 2020-05-02

**Authors:** Jinle Xiong, Xueyu Liang, Lina Zhao, Benny Lo, Jianqing Li, Chengyu Liu

**Affiliations:** 1 School of Instrument Science and Engineering, Southeast University, Nanjing 210096, China; 213162269@seu.edu.cn (J.X.); 213163373@seu.edu.cn (X.L.); zhaolina0808@126.com (L.Z.); ljq@seu.edu.cn (J.L.); 2 The Hamlyn Centre/Department Surgery and Cancer, Imperial College London, London SW7 2AZ, UK; benny.lo@imperial.ac.uk

**Keywords:** cardiovascular time series, congestive heart failure, data refinement, heart rate variability, sample entropy

## Abstract

Due to the wide inter- and intra-individual variability, short-term heart rate variability (HRV) analysis (usually 5 min) might lead to inaccuracy in detecting heart failure. Therefore, RR interval segmentation, which can reflect the individual heart condition, has been a key research challenge for accurate detection of heart failure. Previous studies mainly focus on analyzing the entire 24-h ECG recordings from all individuals in the database which often led to poor detection rate. In this study, we propose a set of data refinement procedures, which can automatically extract heart failure segments and yield better detection of heart failure. The procedures roughly contain three steps: (1) select fast heart rate sequences, (2) apply dynamic time warping (DTW) measure to filter out dissimilar segments, and (3) pick out individuals with large numbers of segments preserved. A physical threshold-based Sample Entropy (SampEn) was applied to distinguish congestive heart failure (CHF) subjects from normal sinus rhythm (NSR) ones, and results using the traditional threshold were also discussed. Experiment on the PhysioNet/MIT RR Interval Databases showed that in SampEn analysis (embedding dimension *m* = 1, tolerance threshold *r* = 12 ms and time series length *N* = 300), the accuracy value after data refinement has increased to 90.46% from 75.07%. Meanwhile, for the proposed procedures, the area under receiver operating characteristic curve (AUC) value has reached 95.73%, which outperforms the original method (i.e., without applying the proposed data refinement procedures) with AUC of 76.83%. The results have shown that our proposed data refinement procedures can significantly improve the accuracy in heart failure detection.

## 1. Introduction

Congestive heart failure (CHF) is a complex clinical syndrome that can result from any structural or functional cardiac or non-cardiac disorder [1]. When it occurs, the ability of the heart to respond to physiological demands for increased cardiac output is impaired [2]. Common factors such as secondary coronary disease, hypertension, valvular disease, myocarditis, diabetes, alcohol excess and obesity can all be the causes for heart failure [3,4]. As approximately 26 million people suffer from CHF worldwide, it is a leading cause of mortality and morbidity [5,6]. Nevertheless, the diagnosis of heart failure is quite challenging [7], which relies on analyzing the patient history and physical examination [8], and clinical examination often fails to detect heart failures [9].

Since the use of ECG for clinical diagnosis, heart rate signals are regarded as a main source of information for CHF diagnosis [10] and much research has studied heart rate dynamics. Heart rate variability (HRV) analyzes the fluctuation in the time interval between consecutive heartbeats [11], which is an indicator of autonomic nervous system modulation [12]. HRV can be evaluated using linear methods, analyzed in the time and frequency domains, as well as non-linear methods [13]. Since a healthy heart’s beat-to-beat fluctuations are best described by mathematical chaos [14], non-linear analysis can identify complex interactions from various mechanisms that are markers of cardiac variability [12], thus providing promising tools for HRV assessment [15]. Among the non-linear measurements, entropy quantifies the regularity of physiological time series and elicits valuable information of the cardiovascular system, thus it is widely used in many research fields [16]. One of the entropy measures that are highly adapted to cardiovascular signal processing is sample entropy (SampEn), which can overcome the bias and self-matching problem of approximate entropy (ApEn) [17].

As a typical application for SampEn in clinical measurement, there have been numerous studies on distinguishing CHF subjects from normal sinus rhythm (NSR) ones [18,19]. In [20], a parametric estimation of SampEn on real RR series from both NSR and CHF subjects was proven to be feasible. SampEn has also been widely chosen as a representative feature in novel CHF detection algorithms based on HRV measures and classifiers such as support vector machine (SVM) [21,22,23,24], which provide effective and computationally efficient tools to automatically diagnose CHF patients. Though diagnosing heart failure with long-term ECG signals (usually 24 h) may lead to accurate results [25,26], some studies have shown that short-term (usually 5 min) ECG recordings could also provide valuable information [27,28], thus the focus of research has recently switched to low-cost, non-invasive, and lightweight classification methods that are based on short-term HRV analysis [22]. Nevertheless, the significant variation in both intra-individual and inter-individual makes it hard to achieve high accuracy. We hypothesized that the selection of certain sequences according to the neuroactivity of the heart from the entire long-term signals might reveal the underlying heart condition of the individual. In addition, instead of processing data of all subjects from the widely used databases, such as the NSR RR interval database and CHF RR interval database at PhysioBank, or simply picking out several subjects to meet specific requirements, it is necessary to have unified standards for selecting high-quality signal segments. Therefore, we divide the subjects into several types based on the evaluation of their quality in CHF detection.

In previous study [29], we introduced a physical threshold-based SampEn to solve the inconsistency issue of traditional SampEn in heart failure diagnosis, and experiments have shown its performance. In this study, we continue to use the physical threshold-based SampEn but for data selection rather than entropy calculation. Experimental results with the traditional threshold are also presented.

In this work, our aim is to improve the accuracy of SampEn in heart failure detection by refining the process of the original RR interval time series. Verification will be performed on NSR and CHF groups to validate the effect of the proposed method. The rest of the paper is organized as follows. Section 2 describes the algorithm of SampEn with physical threshold and the flawed results in the previous study. On that basis, the selection of fast heart rate (HR) sequence is proposed, and another similarity measure, Dynamic Time Warping (DTW), is added to further advance the method. The experiment process and results are presented in Section 3 and Section 4 respectively. Section 5 gives a discussion and conclusion.

## 2. Methods

### 2.1. Physical Threshold-based SampEn

Sample entropy (SampEn) is a non-linear measurement of system complexity, which calculates the negative logarithm of the conditional probability that two sequences within a tolerance *r* for *m* points remain within *r* of each other at the next points [17]. According to previous research, SampEn with physical threshold was taken as baseline algorithms in this study. The calculation of the physical threshold-based SampEn was summarized as follows [17,30]:

For RR segment *x*(*i*) (1 *≤ i ≤ N*), given the parameters *m* and *r*, first form the vector sequence $X_{i}^{m}$:

(1) $$X_{i}^{m}=\left\{x\left(i\right),\;x\left(i+1\right),\cdots ,x\left(i+m-1\right)\right\}\;\;1\;\leq \;i\;\leq \;N-m$$

The vector $X_{i}^{m}$ represents *m* consecutive *x*(*i*) values. Then the distance between $X_{i}^{m}$ and $X_{j}^{m}$ based on the maximum absolute difference is defined as:

(2) $$d_{i,j}^{m}=d\left[X_{i}^{m},X_{j}^{m}\;\right]=\underset{0\;\leq \;k\;\leq \;m-1}{\max}\left|x\left(i+k\right)-x\left(j+k\right)\right|$$

For each $X_{i}^{m}$, denote $B_{i}^{m}$(*r*) as (*N* − *m*)^−1^ times the number of $X_{j}^{m}$ (1 *≤ j ≤ N* − *m*) that meets $d_{i,j}^{m}$ ≤ *r*. Similarly, set $A_{i}^{m}$(*r*) as (*N*−*m*)^−1^ times the number of $X_{j}^{m+1}$ that meets $d_{i,j}^{m+1}$ ≤ *r* for all 1 *≤ j ≤ N* − *m*. Typically, recommended *r* for clinical use is between 0.10 and 0.25 times the standard deviation (SD) of the data [31]. Nevertheless, under certain circumstances, NSR group presented higher SampEn results than those in CHF group when *r* was set to 0.10, while the outcomes reversed as *r* increased to 0.25 [32]. The inverted entropy results make it hard to establish a unified standard to detect CHF subjects with a constant *r* value. To avoid such inconsistency, we proposed a physical threshold as multiple of sampling period, and proved that it is more adaptive to CHF detection than the traditional threshold [29]. Since the signals were sampled at 128 Hz, we regarded sampling period as 8 ms, and set threshold *r* as 1.5 times the sampling period, which equals to 12 ms.

Then SampEn is defined by

(3) $$SampEn=\left(m,\;r,\;N\right)=-ln\left(\sum_{i=1}^{N-m}A_{i}^{m}\left(r\right)/\sum_{i=1}^{N-m}B_{i}^{m}\left(r\right)\right)$$

Herein, we defined another parameter in the calculation of entropy metrics: embedding dimension *m* = 2, aiming to avoid inefficient entropy results caused by a relatively large *m*. We also selected the time series length *N* to be 300 to check the influence of various-size RR interval segments [33], for time series with length of 10*^m^* to 10*^m^*^+1^.

### 2.2. Why SampEn Results of Heart Failure Individuals Resemble Normal Ones

**Figure 1:**
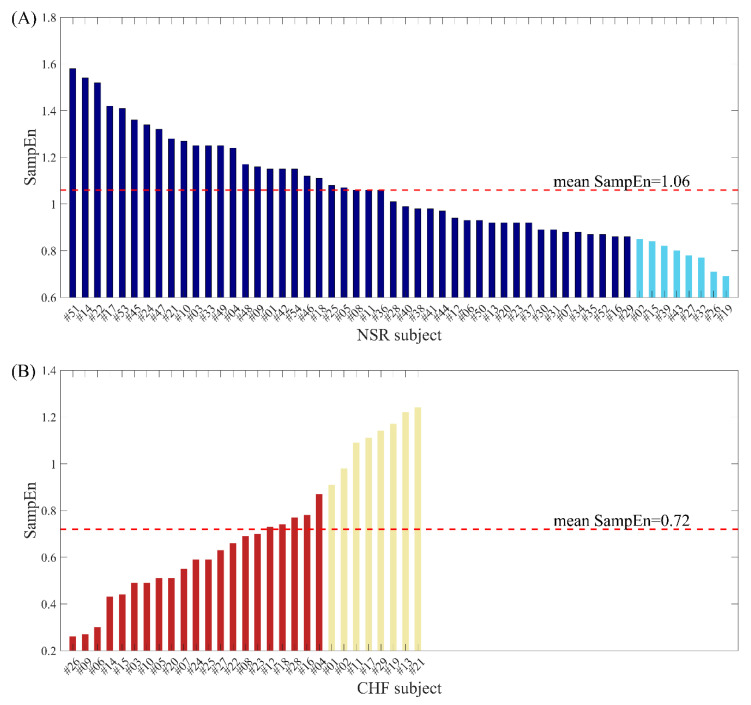
The average SampEn values when using *m* = 1, *r* = 12 ms and *N* = 300 for (**A**) NSR subjects and (**B**) CHF subjects.

In previous study [29], we used physical threshold-based SampEn to distinguish CHF subjects from NSR subjects. As the results have shown, the average SampEn values of CHF group are significantly lower than those of NSR group when using *m* = 1, 2 and *N* = 300, 1000, respectively. Nevertheless, several individuals in CHF group present relative higher entropy results, blurring the boundary between CHF and NSR subjects. Similarly, there are also some NSR individuals that show relatively low entropy values, leading to the confusion in distinguishing these two types. Figure 1 rearranges average SampEn value of each individual using *m* = 1, *r* = 12 ms and *N* = 300 and marks out those outliers for NSR and CHF respectively. Herein, the average SampEn value refers to the mean value of all the SampEn results from each segment of length *N* = 300 within one individual. The eight NSR subjects marked out in light blue in subplot (A) are those who have the smallest SampEn values compared to the rest of the group. Likewise, the eight CHF subjects marked out in pale golden in subplot (B) are those who have the largest SampEn values among the group. Their average SampEn values reveal that some individuals in CHF group are very similar to healthy individuals, while certain NSR subjects can also present heart failure characteristics. All these entropy values would lead to confusion in discriminating CHF subjects from NSR ones, since they make it hard to draw a boundary between the two groups.

**Figure 2:**
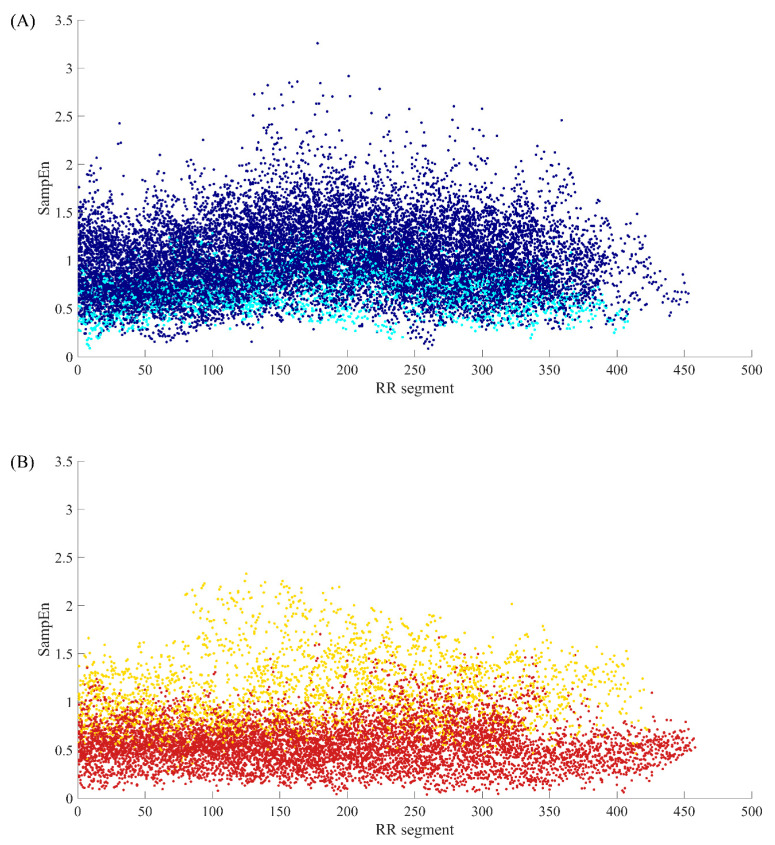
The SampEn distribution of all RR segments when *m* = 1, *r* = 12 ms and *N* = 300 for (**A**) NSR group and (**B**) CHF group.

Figure 2 illustrates the distribution of SampEn values from all RR segments (embedding dimension *m* = 1, threshold *r* = 12 ms, segment length *N* = 300) for both NSR and CHF groups. In the top graph, segments from NSR002, NSR015, NSR019, NSR026, NSR027, NSR032, NSR039 and NSR043 are shown in cyan, while segments from the remaining subjects are represented by dark blue points. We can see that most of the bottom points consist of the cyan ones, which indicates certain segments from the above-mentioned subjects have relatively low entropy values, thus being less characteristic of healthy people. Nevertheless, we should not ignore the fact that some points from these 8 subjects overlap with the dark blue ones, implying that they show no difference with the majority of healthy individuals. In the bottom graph, segments from CHF201, CHF202, CHF211, CHF213, CHF217, CHF219, CHF221 and CHF229 are marked out by gold points, and segments from other subjects are shown as firebrick points. The deviation of these 8 subjects can be clearly observed in this graph. Almost all the points with higher SampEn values are from the aforementioned individuals, which demonstrates their RR interval time series are more similar to the healthy group rather than heart failure one. Meanwhile, these 8 subjects still have some segments that overlap with the majority of CHF group.

**Figure 3:**
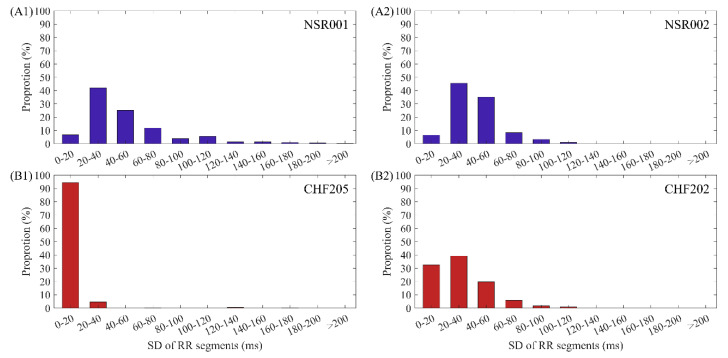
The distribution ranges of SD values using *N* = 300 for (**A1**) NSR001, (**A2**) NSR002, (**B1**) CHF205 and (**B2**) CHF202.

To further explore the causes of these confusing entropy values in both NSR and CHF groups, we chose several representative individuals and analyzed their RR interval series. From Figure 1, we can see that the SampEn values for NSR001 and CHF205 indicate that they are typical individuals of these two groups, while the entropy values of some subjects are more similar to the groups opposite to their physical labels, such as NSR002 and CHF202. Therefore, we selected these subjects for comparison. Figure 3 gives the distribution ranges of SD of RR interval series from the aforementioned subjects using *N* = 300. In (A1) and (B1), we came to the conclusion that the SD values of RR segments for typical CHF subject are relatively small, assembling in the area under 40 ms, while the typical NSR subject shows a dispersed distribution. However, in (A2), SD values between 20 ms and 60 ms appear more often, and there are no SD values beyond 120 ms, implying that the fluctuation in the RR time series of NSR002 is slightly lower than NSR001. Meanwhile, in (B2), the distribution of SD values from CHF202 is obviously more dispersed and less representative of heart failure subjects than CHF205.

From the analysis above, we have found that certain individuals in NSR and CHF groups present SampEn values that deviate from the majority significantly, influencing the average values of the whole group. This is because the RR time series of these individuals are quite different from others, and the SampEn results of many of their RR segments appear beyond the dividing line of healthy group and heart failure group, leading to difficulty in identifying CHF subjects.

In our previous study [29], we used almost the whole RR time series from one subject to calculate SampEn. However, we found that this approach may not be rational due to the fact that the RR interval series from a healthy subject might present similar segments to those with heart failure, while a heart failure subject does not show symptoms at every moment. Analyzing all the RR segments from one subject would include those interrupting periods, thus affecting the average SampEn value and causing confusion in CHF detection. Therefore, it is necessary to select proper RR segments for SampEn calculation.

### 2.3. Discrimination of fast HR Sequences

When using the whole RR interval time series to calculate SampEn, the signals contain various heart health status of the individuals in nearly 24 h. That means these subjects may experience states of motion, resting or sleeping during the observation time, introducing exercise heart rate as well as resting heart rate in the time series. The great variability in heart rate during a 24 h period is well known from earlier studies, and it has been proved that leisure-time physical activity can cause statistically significant effect on the heart rate level over a 24 h period [34]. Moreover, CHF subjects can only have limited physical activities, especially those diagnosed as NYHA class II, III and IV [35]. As a consequence, these subjects would stay in resting state for the majority of the time in the 24 h period. Since the overall lengths of the time series for both NSR and CHF groups are approximately the same, and heart failure people are often not able to exercise, the proportions of resting state and physical activity would vary from CHF to NSR, even from different individuals within the same group. The comparison of SampEn using the whole 24 h records would be unreasonable, as we might compare the kinematic period of one individual with the motionless period of another one unconsciously.

To further explore the variable periods of RR interval time series in 24 h, we tried to extract RR segments that can reflect the internal characteristics of each individual’s heart condition even if there are influences from personal, environmental, or social factors. Previous studies have pointed out that, heart failure subjects do not present corresponding symptoms sometimes [36], and cardiac arrhythmia might appear in healthy people during a relative long observation period. In order to select segments reflecting an individual’s heart condition, we ought to consider how to capture the symptoms of heart failure when analyzing the RR time series.

CHF is associated with prominent alterations in the autonomic control of the cardiovascular system. Under the influence of various pathogenic factors, impaired contraction or diastolic function of the heart leads to low cardiac output and a decreased effective blood volume. To maintain the stability of the body, compensation appears, which involves activation of systems controlling body fluids, cardiac output and systemic blood pressure [37]. During this mechanism, CHF is usually accompanied by cardiovascular signs of an increased sympathetic and a decreased parasympathetic activity [38], as well as the activation of other neurohormonal systems [39]. One result of the compensation is the increase in heart rate, causing the decrease of mean RR intervals in certain periods [40,41]. Moreover, rapid ventricular pacing would induce CHF sometimes, and ventricular tachycardia and fibrillation occur in some severe heart failure patients [42]. All these might contribute to relative short RR intervals.

As healthy people are more likely to have elevated heart rate due to physical activity while heart failure one’s present fast heart rate due to symptoms, it is clear that the causes for sudden decreases in RR interval values are fundamentally different for NSR and CHF groups. For this reason, we postulated RR intervals with lower values would be more representative for individuals, and basing on this hypothesis, we preferred to select RR segments that show a sudden drop during the entire time series as the intrinsic sequences, which reflect the characteristics of each individual’s heart condition.

**Figure 4:**
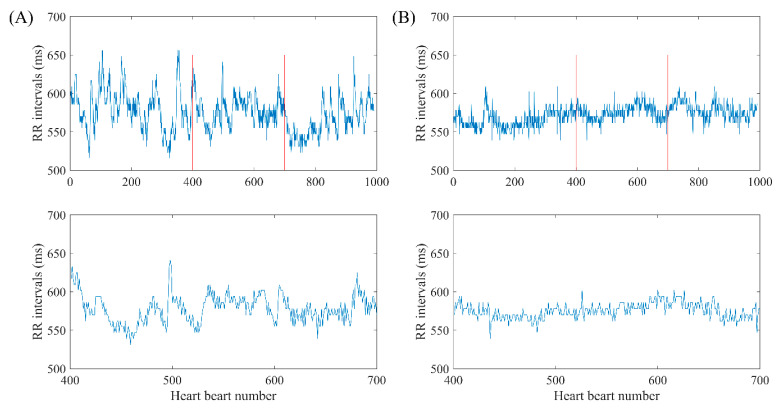
Selection of fast HR sequence with length *N* = 1000 and 300 respectively for (**A**) NSR001 and (**B**) CHF205.

To explain the selection of our fast HR sequences in detail, we chose RR segments from two typical subjects’ signals, NSR001 and CHF205, to compare their differences. The RR segments for both subjects contain 1000 heart beats, and the mean RR interval is 575 ms for the NSR subject and 572 ms for the CHF one, which both represent a relative faster heart rate than the remaining parts in their time series. In Figure 4, the top ones give an overview of the whole intrinsic sequence, and the bottom ones magnify 300 heart beats to show the difference in morphology. We can see from the subplots that the sequences of these two individuals are completely different in their shapes, and the sequence from NSR001 has larger variation though the two sequences share similar mean RR interval values. In fact, SD values of the sequences are 27.9 ms and 13.6 ms for NSR001 and CHF205 respectively, which can be used to distinguish the heart failure subject from healthy one. Therefore, selecting fast HR sequences before analyzing SampEn seems to be feasible.

### 2.4. Dynamic Time Warping

Compared with traditional data mining tasks, RR interval time series have their own characteristics, thus it would be unreasonable to simply regard them as high-dimensional data and apply machine learning algorithms for classification. In time series data mining research, one of the core problems is the similarity measurement of time series, which would also decide clustering and classification results.

Besides distance-based similarity in entropy measurements, we would like to search for similarity measures that are not limited to time series with the same baseline, scale, and length. One algorithm that is widely adopted for processing misaligned time series is dynamic time warping (DTW). This is a nonlinear distance measure that originated in the speech recognition community and it maps one time series onto another. It has been used in a wide range of applications, from medicine to robotics. The calculation of DTW is summarized as follows [43]:

Assume we have a time series $X\;=\left\{x_{1},\;x_{2},\;\ldots ,\;x_{m}\right\}$ of length *m*, and another time series $Y\;=\left\{y_{1},\;y_{2},\;\ldots ,\;y_{k}\right\}$ of length *k*. The warping path $W\;=\left\{w_{1},\;w_{2},\;\ldots ,\;w_{N}\right\}$, where max(k,m) ≤ N ≤ k+m−1, is found using dynamic programming to compute the cumulative distance CUM_DIST(i,j):

(4) $$\begin{array}{c} CUM\_DIST\left(i,j\right)=dist\left(i,j\right)+\min\{CUM\_DIST\left(i-1,j-1\right),\\ CUM\_DIST\left(i-1,j\right),\;\;CUM\_DIST\left(i,j-1\right)\} \end{array}$$

where the k×m matrix dist is defined such that the (i,j) element contains the distance of $x_{i}$ and $y_{j}$. The warping path satisfies the following conditions:

⬤ It starts and finishes in diagonally opposite corner sides of the matrix.
⬤ The cells in the warping path have to be adjacent (including diagonally).
⬤ The points in W have to be placed monotonically in space.

Computation of Equation (4) leads to the derivation of an optimum warping path, which minimizes the following warping cost:

(5) $$COST=\sqrt{\sum_{n=1}^{N}w_{n}}/N$$

**Figure 5:**
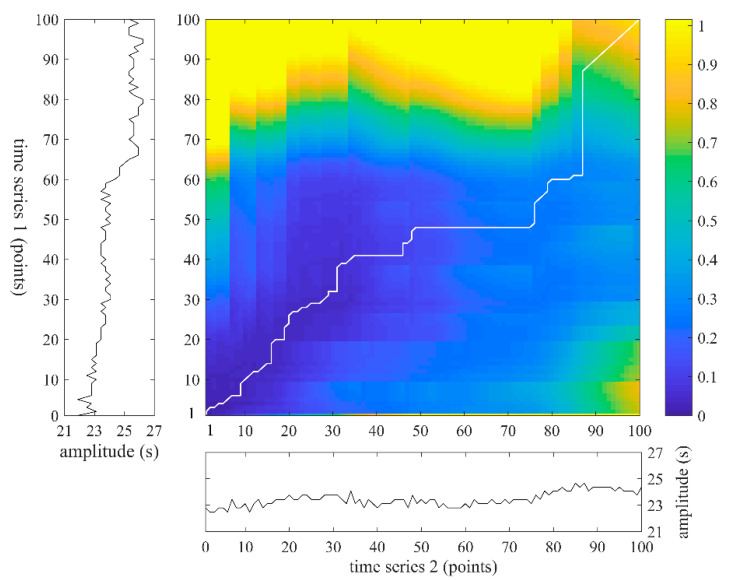
An example of warping path using two time series which both contain 100 points.

Figure 5 gives an example of warping path using two concrete time series. Herein, dist is a 100×100 matrix, and similarity is presented by color bar. The smaller the value is, the more similar the two sequences are. The optimum warping path is marked out by a white line in the color map, which has the minimal warping cost. Herein, the minimal cost value is 60.76.

To assure the sequences we select possess unified features, similarity search of time series is performed using DTW, and we can exclude those sequences that are different from the remaining parts by analyzing the DTW costs.

## 3. Data and Experiment

### 3.1. Data

All data used is from the RR Interval Databases from http://www.physionet.org [44], a free-access, on-line archive of physiological signals. The NSR RR Interval Database was used as the non-pathological and control group data, which includes 54 long-term RR interval recordings of subjects in normal sinus rhythm aged 29 to 76. The CHF RR Interval Database was used as the pathological group data. This database includes 29 long-term RR interval recordings of subjects aged 34 to 79, with congestive heart failure (NYHA classes I, II, and III). Each of the long-term RR interval recordings is a 24-h recording including both daytime and night-time. The Holter ECG measurement was taken for both NSR and CHF groups under the similar level of physical activity. The original ECG signals were sampled at 128 Hz, and the beat annotations were obtained by automated analysis with a manual review and correction.

### 3.2. Experiment Scheme

**Figure 6:**
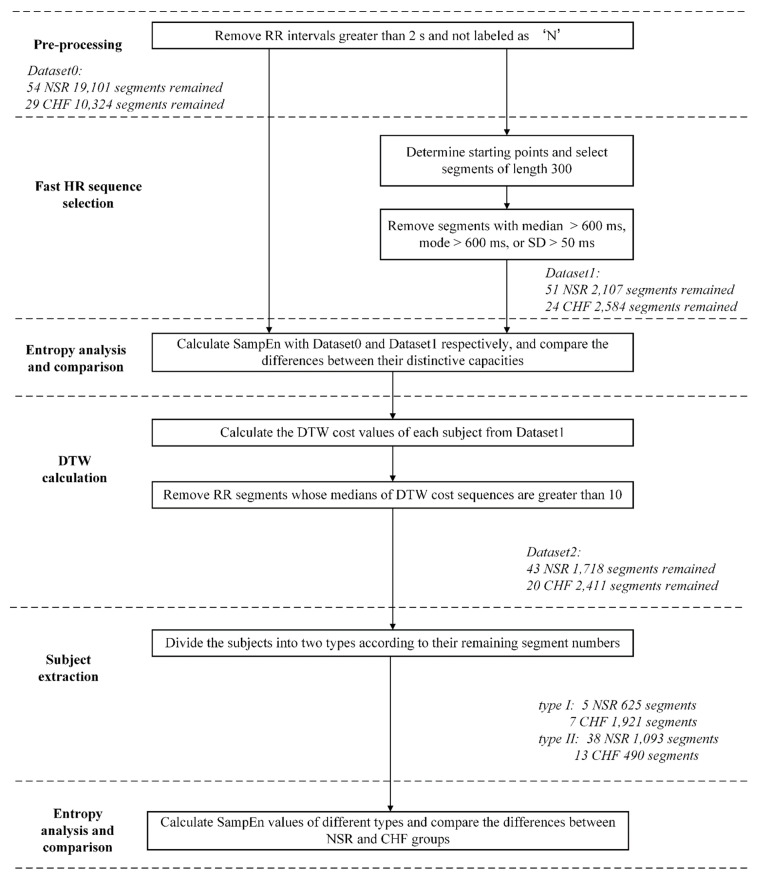
Block diagram of the proposed procedure. NSR: normal sinus rhythm, CHF: congestive heart failure, SD: standard deviation, DTW: dynamic time warping. Herein, segments imply RR segments of length 300.

Figure 6 shows the block diagram of the analytical procedure used in the present study. This procedure contains six major steps: (1) pre-processing for each RR interval recording; (2) select RR sequences as fast HR sequences; (3) calculate SampEn of different datasets and make a comparison to determine whether the selection of certain parts in the time series is as effective as all segments preserved in entropy calculation; (4) calculate the DTW cost values of each two segments within one certain subject and exclude segments that are dissimilar with others; (5) analyze the remaining segments of each subject and classify the subjects of both NSR and CHF groups into several types; (6) calculate and compare the entropy results of the corresponding types in both NSR and CHF groups, and determine whether the extraction of certain subjects with high-quality RR interval series would improve the results for heart failure detection.

In Step (1), the RR intervals greater than 2s were firstly removed from the raw RR interval recordings to minimize the influence from artifacts. For each beat in the raw ECG signals, it was annotated as a normal (denoted as ‘N’) or abnormal heartbeat. The abnormal heartbeats were usually caused by the ectopic beats such as supra-ventricular ectopic beats or ventricular ectopic beats, depending on the localization of the ectopic focus. The RR intervals formed from the abnormal heartbeats could confound the entropy analysis of HRV [45]. So, these RR intervals were then removed from the RR interval recordings.

In Step (2), fast HR sequence that could reflect a period with relative fast heart rate was selected progressively. To begin with, we needed to determine a starting point according to the RR interval. We set the threshold value as 600 ms according to the analysis of overall data distribution, and only considered points no greater than this threshold, implying that there was a fast heart rate at that instance. Then, we choose the RR intervals after it and form a segment of 300 points. Next, we checked the median number, mode number and standard deviation of the segment successively, and preserved those segments with median number no greater than 600 ms, mode number no greater than 600 ms and standard deviation smaller than 50 ms. These conditions were set empirically and could guarantee that the segment we selected would maintain a relative stable state, while the heart rate is still high. After the selection of fast HR sequences, we could get part of the time series from each subject data, and the middle part of Table 1 gives an overview of the total numbers of RR segments for both NSR and CHF groups when setting *N* = 300, in contrast with the original dataset in the upper part of Table 1. The time series length of 300 has been proved to be stable in the analysis according to our previous study [29]. The amount of RR intervals engaged in entropy calculation was approximately 16% of the original dataset after removing unwanted periods. It is worth stating that no sequence from NSR002, NSR003, NSR033, CHF203, CHF204, CHF208, CHF216 and CHF219 was obtained through this selection. Thus, these subjects’ data did not contain sequences that met our requirement, and we excluded them in the following analysis.

In Step (3), SampEn was used to calculate the entropy values for the two datasets under the different parameter settings: embedding dimension *m* was set as 1 and 2, and physical threshold *r* was set as 12 ms. Since the length of the fast HR sequence selected above was set as 300 points, we used segment length *N* = 300 to analyze SampEn. For each RR segment, we removed the RR intervals outside the 99% confidence interval (CI), (i.e., ± 3 × SD). Then, the entropy results were compared between the NSR and CHF groups under the certain parameter combinations. The comparison aimed to explore whether the selection of fast HR sequences for analysis could still be effective in distinguishing the CHF patients from the NSR subjects.

In Step (4), we used another similarity measure, DTW, to assess the relation between two RR segments. Each two RR segments of one subject were chosen to calculate the minimal DTW cost. Then we could construct a n × n matrix consisted of these cost values, where n represents the number of RR segments of the subject preserved after fast HR sequence selection. Then, we rearranged the values of each column from small to large, recorded the median number of each column and compared it with 10. In this calculation, the larger the DTW cost value is, the more dissimilar the two segments are. To filter out segments that do not share uniformed characteristics in sequence morphology with others, we set the threshold as 10 empirically, and discarded segments whose median numbers are greater than the threshold, which implied that they are different from the majority. This process was computationally intensive, as we had to calculate hundreds of times for each subject using DTW measure.

In Step (5), we counted the numbers of the remaining RR segments of each subject after DTW processing. Based on the outcomes, we set 90 as the boundary and divided the overall subjects into two different types. For the sake of brevity, we called the subjects with segment numbers no smaller than 90 as type I, and the remaining ones who have less than 90 segments as type II. 5 NSR (NSR005, NSR020, NSR025, NSR029 and NSR054) subjects and 7 CHF (CHF205, CHF206, CHF210, CHF215, CHF220, CHF224 and CHF226) subjects were contained in type I, while the rest of the subjects were regarded as type II. The lower part of Table 1 shows the segments left both after DTW calculation and subject extraction. According to our proposition, the more RR segments preserved, the better heart failure detection. No matter one individual is NSR or CHF, more RR segments remain after the selections means that heart failure detection from data of such individuals would result in higher sensitivity, specificity as well as accuracy.

In Step (6), we evaluated the efficacy of our extraction via SampEn using different combinations of parameters (*m* = 1 and 2, *r* = 12 ms, and *N* = 300). Only two corresponding types that possessed similar segment numbers were compared, as we tried to explore whether the secondary screening would give a reasonable assessment of the time series from each individual.

In the diagram, we name the original dataset as Dataset0, the dataset after fast HR sequence selection as Dataset1, and the one after DTW processing as Dataset2. In addition, as Dataset2 has been divided into two types in Section 4.2, we call the subjects with segment numbers no smaller than 90 as type I, and the remaining ones who have less than 90 segments as type II for brevity.

### 3.3. Statistical Analysis

In SampEn calculation, for each RR segment with length of *N* = 300, embedding dimension *m* = 1 and 2, and physical threshold *r* = 12 ms were applied for both original dataset and the dataset with fast HR sequence selected. The overall mean and SD values of these two methods were calculated across all RR interval recordings, separately for the NSR and CHF groups. The parametric test, Student’s *t*-test, was used to test the statistical difference between the two groups. All statistical analyses were performed using the MATLAB software (Version R2017a, The MathWorks, Natick, MA, USA). Statistical significance was set a priori at *P* < 0.01.

In the following analysis, entropy values on one side of a threshold *c* were labelled as CHF while values on the other side of *c* were labelled as NSR. Classifier accuracy was assessed via the following performance metrics:

- Sensitivity: Se = TP/(TP+FN)
- Specificity: Sp = TN/(TN+FP)
- Accuracy: Acc =(TP+TN)/(TP+FP+FN+TN)

where TP, TN, FP and FN are the numbers of true positives, true negatives, false positives and false negatives respectively.

Moreover, the receiving operator curve (ROC) and area under the curve (AUC) were used to evaluate the effectiveness of the proposed method in CHF detection. The ROC curve is a plot (*Se*) versus (1-*Sp*) for many possible values of *c*, which varied from the minimum to the maximum of the entropy outputs, with a step of 1% of the range. Another metric for assessing ROC curves, the Youden index (*J*), was also calculated as:

- $\;J\;=\underset{c}{\max}\left\{Se\left(c\right)+Sp\left(c\right)-1\right\}$

At the optimal cut-point c*, J is maximized, and the classifier equally weighs sensitivity and specificity. The aforementioned performance metrics of *Se*, *Sp*, and *Acc* were given at the point of c*. Realizing the importance of the index’s effectiveness for accepting or excluding patients under high probability, we also calculated the performance metrics at the setting of cut-point *c* for *Se* > 99% and *Sp* > 99%, respectively [46].

## 4. Results

### 4.1. Results of Fast HR Sequences Selection and DTW Processing

**Figure 7:**
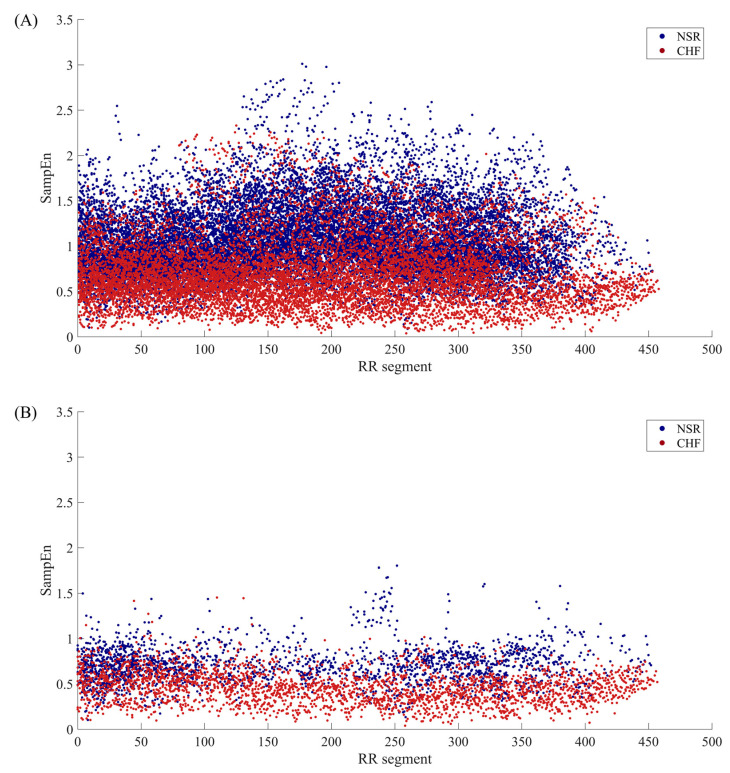
The SampEn distribution of RR segments involved in calculation when *m* = 1, *r* = 12 ms and *N* = 300 for (**A**) Datast0 and (**B**) Dataset1.

**Figure 8:**
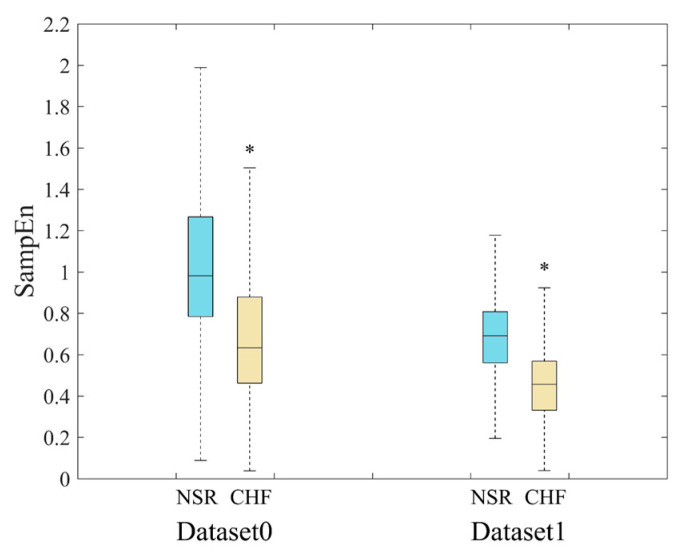
Distribution ranges of SampEn between NSR and CHF groups when *m* = 1, *r* = 12 ms and *N* = 300 for Datast0 and Dataset1. *’means there is a significant difference between NSR and CHF groups.

SampEn results of RR segments from both original method and the proposed method are shown in Figure 7. The distribution of all segments from original dataset is presented in the top graph, and we can see that there is a quite large overlapping area in the middle of the point set, which makes it difficult to distinguish CHF subjects from NSR ones. In contrast, the RR segments selected through our proposed method are displayed in the bottom graph according to their positions in the entire time series of a certain subject. The distribution of these points implies that the junctional area is diminished, and less points are involved in SampEn calculation, especially for NSR group. This also indicates that CHF subjects possess more fast HR sequences than NSR group, though the total RR interval number of the former is rather small. Figure 8 presents the distribution ranges of SampEn under the same circumstances. It is obvious that the overlapping part between NSR and CHF groups in Dataset0 has almost disappeared in Dataset1.

The SampEn values as well as statistical significance were calculated and presented in Table 2. The parameter combinations included *m* = 1 and 2, and *r* = 12 ms, while *N* was set to 300, due to the limited length of the sequences selected. In the first part of Table 2, SampEn results from Dataset0 are displayed for comparison. We can see that all the entropy values from NSR group are consistently higher than those from CHF group, and there is a significant difference between the two groups with each parameter combination. This indicates that the original method is able to distinguish CHF from NSR subjects. In comparison, after the selection of fast HR sequences, segments with relatively small as well as stable RR intervals were preserved, thus the average SampEn values of each group decreased. Furthermore, as the second part of Table 2 shows, the SD values tend to be smaller in our proposed method, and *p* values are also much smaller, implying larger difference between the two groups. In the third part of the table, results of Dataset2 are presented. Herein, we have to state that Dataset2 in this section refers to the entire dataset without distinguishing between type I and type II. The DTW processing has widened the gap between NSR and CHF groups, and SD values become even smaller. When applying *m* = 1, the difference between the mean values of NSR and CHF groups is 0.24 in Dataset1, and the same value is 0.26 in Dataset2, which has increased by 8%. Likewise, when using *m* = 2, the difference between the mean values of the two groups is 0.21 in Dataset1, and the same value is 0.23 in Dataset2, which has increased by nearly 10%. It is worth noting that CHF subjects are less vulnerable to be changed by DTW than NSR ones, and *p* values do not seem to improve much under the circumstances. This might be attributed to the fact that the filtering effect of DTW varies individually. Although the gap between the average SampEn values of the two groups has become larger, some subjects’ data have preserved segments whose features disaccord with their labels, leading to slightly declining statistical significance in overall analysis. Thus, we would explore the filtering effect of DTW processing on different subjects in the later part.

Table 3 summarizes the classifier performance metrics of the three datasets at three settings of cut-point *c* values: optimal cut-point c* (equally weighs sensitivity and specificity), cut-point *c* for highly weighting sensitivity (*Se* > 99%) and highly weighting specificity (*Sp* > 99%). For optimal cut-point c* that equally weighs sensitivity and specificity, indexes *J*, *Se*, *Sp* and *Acc* are 43.35%, 60.31%, 83.05% and 75.07% respectively for SampEn calculation using Dataset0. As for Dataset1, the same indexes are 49.14%, 75.39%, 73.75% and 74.65% respectively. Although the *Acc* value of the former is slightly higher than the latter, SampEn analysis with proposed dataset has a preferable *J* value, indicating it presents better balance between sensitivity and specificity. Moreover, the same indices are 53.83%, 79.10%, 74.74% and 77.28% for SampEn analysis using Dataset2. We can see that all these values have improved compared to those of Dataset1, thus DTW processing can optimized the results indeed.

For highly weighting sensitivity (*Se* > 99%), we can see that *J*, *Sp* and *Acc* values of Dataset1 are 9.71%, 10.68% and 59.35% respectively, which are better than those from Dataset0. Meanwhile, as the results of Dataset2 show, the DTW screening has increased the indices by approximately 2%.

For highly weighting specificity (*Sp* > 99%), the results of SampEn using Dataset0 outperform both Dataset1 and Dataset2, especially in *Acc* value. This might attribute to the fact that our selection has changed the composition of the overall data. In Dataset1 and Dataset2, CHF segment number is a little higher than the NSR one, while the number of NSR segments is almost two times of the CHF one in Dataset0. Thus, SampEn calculation using the last two datasets would show relatively poor performance when specificity weighs high.

**Figure 9:**
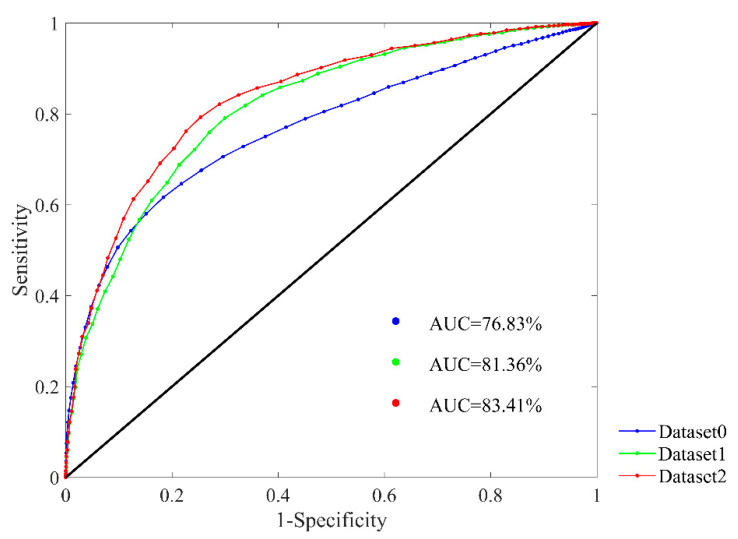
ROC curve plots with AUC values in the RR Interval Databases for classifying NSR and CHF subjects. SampEn results using Dataset0, Dataset1 and Dataset2 are presented respectively. Parameter combination of *m* = 1, *r* = 12 ms and *N* = 300 was used.

Figure 9 presents the ROC plots and AUC values of Dataset0, Dataset1 and Dataset2 respectively. The parameter combination was set as *m* = 1, *r* = 12 ms and *N* = 300. In the figure, the AUC values are 76.83%, 81.36% and 83.41% respectively for the three datasets. The results reveal that after fast HR sequence selection, the prediction outcomes of SampEn would be more appreciable, and the DTW processing has further improved the outcomes.

### 4.2. Results of Subject Filtering

The proportion that each subject’s segments accounted for in the entire Database2 varied greatly. Some individuals’ data had hundreds of segments, while others had dozens of segments, or some even had no segment left after our selection. This implies the quality of time series from different individuals presents unevenness in heart failure detection. Some subjects’ RR segments have distinctive features in accordance with their classification labels, while others show resemblance with the opposite group. Thus, we proposed to filter out the subjects that possess strong intrinsic characteristics in their heart rate signals through the segment numbers they left after fast HR sequence selection. Based on the analysis of segment number distribution, we chose 90 as our boundary, and divided subjects into two types with respect to their segment numbers. Subjects with segment numbers no smaller than 90 were named as type I, and the remaining ones with less than 90 segments were named as type II. The segments from type I subjects have taken a large share in Dataset2, so these subjects have high-quality signals and are supposed to perform better in SampEn analysis. As for type II subjects, their poor-quality signals led to insufficient segments left, and these might be unable to present subjects’ characteristics thoroughly in later discrimination, thus we assumed that their performance in SampEn analysis would be inferior.

In our proposition, through the DTW analysis, the overall results would be improved, as segments that satisfy the magnitude conditions, but have rather different patterns, are removed. To further explore its influence, we made a contrast between the results with and without DTW processing in addition to subject filtering, which means we implemented subject filtering both on Dataset1 and Dataset2 to compare the differences. One thing that requires notice is that three NSR subjects (NSR019, NSR050 and NSR052) in Dataset1 changed from type I to type II after DTW processing, as their remaining segments were less than 90 after this screening.

**Figure 10:**
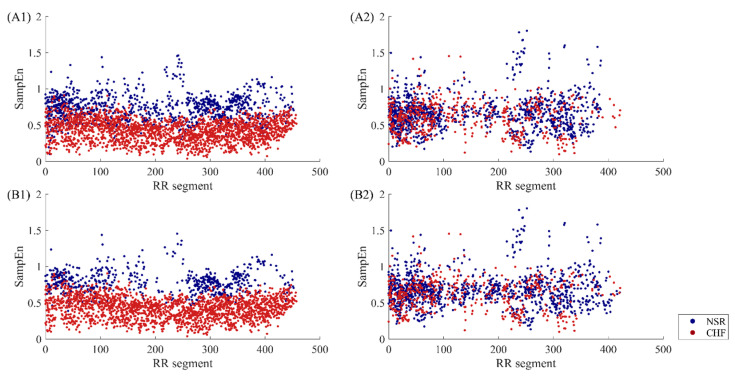
The SampEn distribution of RR segments involved in calculation before and after DTW processing when *m* = 1, *r* = 12 ms and *N* = 300. The top two sub-figures (**A1**, **A2**) show type I subjects and type II subjects respectively before DTW. The bottom two sub-figures (**B1**, **B2**) show type I subjects and type II subjects respectively after DTW.

Figure 10 presents SampEn distribution of RR segments before and after DTW filtering. Sub-figures (A1) and (A2) give a comparison of these two types before using DTW measure. In fact, the two figures are a decomposition of Figure 7B, as (A1) represents subjects that have distinctive features, while (A2) contains the rest of the subjects that are hard to distinguish in heart failure detection. We can see from the figures that the overlapping area in (A1) is smaller, while the points in (A2) still overlap with each other. Therefore, the proposed classification of subjects based on segment number works intuitively. After the DTW processing, sub-figure (B1) indicates that some points of the junctional area are removed, thus the discrimination results are improved. Nevertheless, this secondary screening seems to have less effect on individuals of type II. The distribution of points in (B2) do not change much compared with those in (A2). So, segments from type II subjects show greater variability in their patterns, hence they are less sensitive to DTW measure.

**Figure 11:**
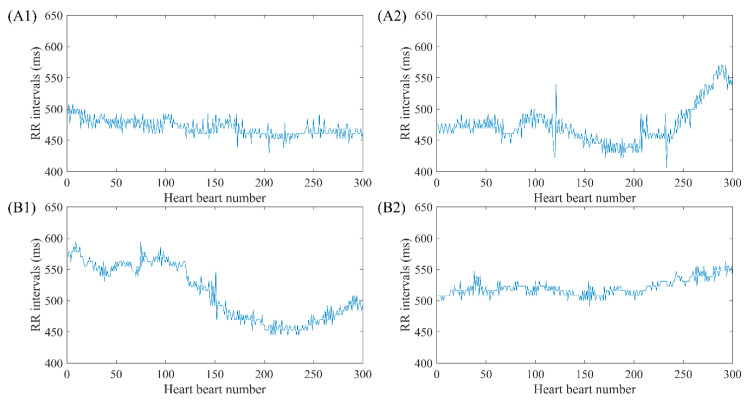
Examples of RR segments removed and preserved after DTW processing for NSR005 and CHF224. The top two sub-figures (**A1**,**A2**) show two segments from NSR005 removed and preserved respectively after DTW, while bottom sub-figures (**B1**,**B2**) show two segments from CHF224 removed and preserved respectively after DTW. Herein, segment length *N* was fixed as 300.

To demonstrate the effect of DTW processing in detail, two subjects from type I, NSR005 and CHF224 were used as examples. In the upper part of Figure 11, two segments from NSR005 are presented in sub-figures (A1) and (A2), and their average RR intervals are quite similar, which are 471 ms and 474 ms respectively. When using *m* = 1, *r* = 12 ms and *N* = 300 for SampEn calculation, the overall mean value of the subject before DTW processing is 0.79, and entropy value of the segment in (A1) is 0.63, while entropy value of the segment in (A2) is 0.80. Since the SampEn result of the segment in (A1) is below the average, it is reasonable to remove it from the whole dataset. Meanwhile, the preservation of the segment in (A2) also makes sense. Likewise, the two segments from CHF224 presented in sub-figures (B1) and (B2) also possess similar average RR intervals, which are 512 ms and 521 ms respectively. When using *m* = 1, *r* = 12 ms and *N* = 300 for SampEn calculation, the overall mean value of the subject before DTW processing is 0.54, and entropy value of the segment in (B1) is 0.65, while entropy value of the segment in (B2) is 0.45. The removal of the segment in (B1) as well as the preservation of the segment in (B2) via DTW processing has improved the entropy result of the individual. Moreover, it is worth noticing that the segments removed in NSR subjects are similar to those preserved in CHF subjects, while the segments removed in CHF subjects resemble those preserved in NSR subjects. This is because DTW measure filters out segments that do not show unified features with the remaining part, and for those typical subjects in type I, we could assume that the unified features for NSR and CHF are just the opposite. Thus, the DTW processing would lead to different results for NSR and CHF subjects.

**Figure 12:**
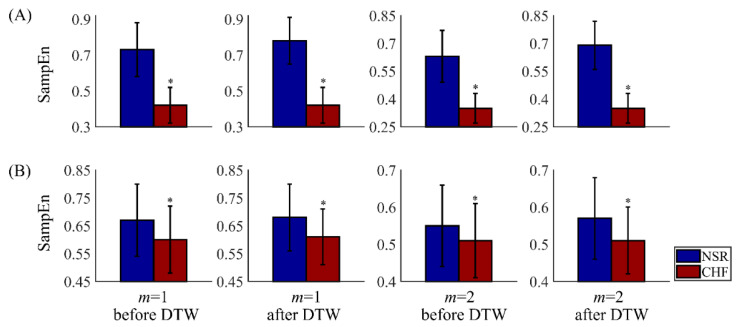
Results of SampEn before and after DTW processing for NSR and CHF groups using *m* = 1 and 2, *r* = 12 ms, and *N* = 300. Herein, subjects are divided into two types: type I in (**A**) with segments no less than 90, and type II in (**B**) with segments less than 90. The symbol ‘*’ means statistical significance *p* < 0.01.

Figure 12 presents the overall SampEn results before and after DTW processing from different combinations of parameters. Physical thresholds *r* = 12 ms and two embedding dimension 1 and 2 were applied here. The segment length was fixed as 300. Comparing the entropy values of type I in (A) and type II in (B), we find that NSR individuals of type II have smaller SampEn values than those from type I, while CHF individuals of type II have larger SampEn values than those from type I. Thus, the classification can distinguish subjects with high-quality time series from those with poor-quality ones in heart failure detection.

In subplot (A), results of type I subjects are displayed, and SampEn values of NSR group are obviously larger than CHF ones. After DTW processing, the gap between NSR and CHF groups has been widened, as the overall SampEn values of NSR have increased to some degree, while the values of CHF stay the same. Since the difference between the two groups becomes bigger, it would be easier to discriminate CHF subjects from NSR ones. Therefore, secondary screening via DTW has been proved to improve SampEn results. Besides, we have to claim that CHF subjects of type I are less sensitive to this measure. This might be attributed to the fact that the patterns of CHF segments from the same individual present less variability and they are regarded as similar to each other through DTW measure.

In subplot (B), results of type II subjects are shown, where SampEn values of NSR and CHF groups are rather similar. Though these outcomes have statistical significance, the *p* values of type II have huge differences in the order of magnitude with those of type I. Moreover, the effect of filtering using DTW is rather limited among type II subjects. Although the difference between the two groups has been amplified slightly, it is still very hard to classify the subjects correctly as their sequences are irregular and disorder in essence. Thus, it is necessary to pick out subjects with distinctive features corresponding to their physical labels from the remaining ones.

Sensitivity, specificity and accuracy results of SampEn before and after DTW processing are shown in Table 4. The results are evaluated at three different cut-point *c* values: optimal cut-point c* (equally weighs sensitivity and specificity), cut-point *c* for highly weighting sensitivity (*Se* > 99%) and highly weighting specificity (*Sp* > 99%). From the first part of the table, which equally weighs sensitivity and specificity are shown, we can see that before DTW processing, the *J*, *Se* and *Acc* values of type I are 66.49%, 88.26% and 84.85% respectively, and the same values of type II are 10.24%, 20.86% and 65.86% respectively. This means classification through SampEn using subjects from type I can achieve reasonable outcomes. Nevertheless, since the NSR segments greatly overweighs CHF ones in type II, its *Sp* value is approximately 11% higher than type I. As for the evaluation after DTW processing, metrics for both types have been improved, especially for type I, whose *J*, *Se*, *Sp* and *Acc* values have reached 78.93%, 91.41%, 87.52% and 90.46% respectively.

For highly weighting sensitivity (*Se* > 99%) in the second part of the table, subjects of type I resulted in significantly larger values in *Sp* and *Acc* than those from type II both before and after DTW processing. For type I, the results of specificity and accuracy have increased from 47.65% and 81.56% to 59.04% and 89.20% respectively, indicating that the application of DTW has improved the classification outcomes. Meanwhile, the corresponding values of type II have decreased slightly after DTW processing.

For highly weighting specificity (*Sp* > 99%) in the last part of the table, the sensitivity value of type I has risen from 32.96% to 61.89% after DTW processing, which is consistently higher than that of type II. As for the accuracy results before DTW processing, type I and type II achieves 55.41% and 66.17% respectively. However, after filtering of DTW, the *Acc* value of type I has reached 71.01%, slightly better than the result of type II.

Since the subjects of type II lower the overall results, we could regard them as poorly effective individuals in the original database, and the filtering by DTW still has a slight effect on these individuals. Another thing that requires notice is that the proportion of data for analysis has been changed. For type I, the NSR segments are only half of the CHF ones in quantity, while for type II, the NSR segments are almost two times of the CHF ones.

**Figure 13:**
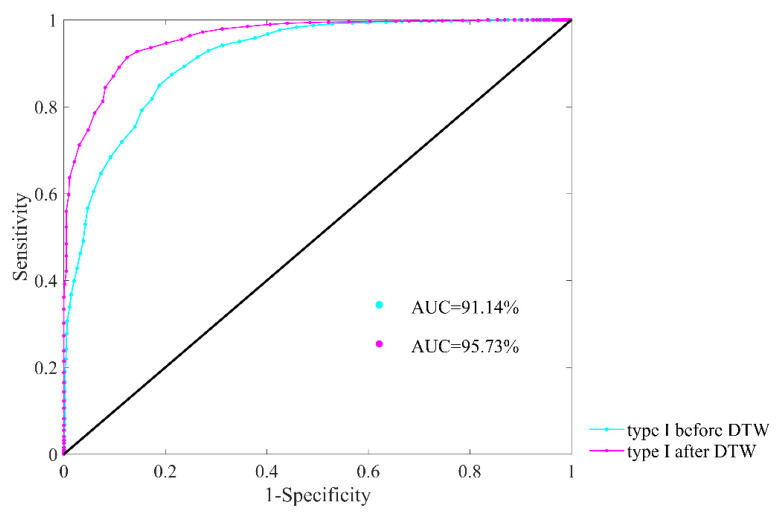
ROC curve plots with AUC values of type I subjects for heart failure detection before and after DTW processing. Herein, embedding dimension *m* was set as 1, physical threshold *r* = 12 ms, and segment length *N* = 300.

Figure 13 presents the ROC plots and AUC values of type I before and after DTW processing with parameter combination of *m* = 1, *r* = 12 ms and *N* = 300. The AUC value is 91.14% for type I before DTW processing, while it has increased to 95.73% after DTW processing. Therefore, the extraction of type I subjects works well in heart failure detection, and the DTW processing leads to even better result. Since the results of type II subjects are rather limited, we have omitted them in this paper.

**Figure 14:**
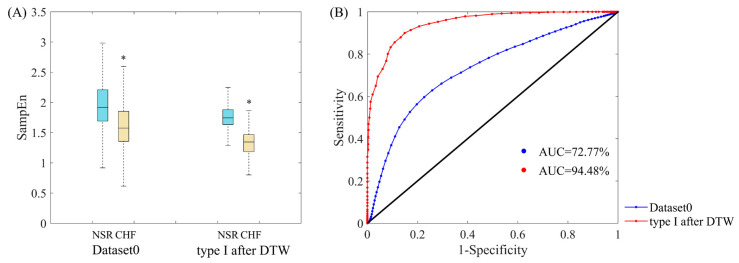
(**A**) Distribution ranges of SampEn with traditional threshold between NSR and CHF groups for Datast0 and type I after DTW. ‘*’means there is a significant difference between NSR and CHF groups. (**B**) ROC curve plots with AUC values of Dataset0 and type I subjects after DTW processing for heart failure detection. Herein, embedding dimension *m* was set as 1, traditional threshold *r* = 0.10, and segment length *N* = 300.

We also made a comparison between original dataset and the data after refinement using SampEn with traditional threshold, aiming to verify this refinement process still works well with recommended *r* (i.e., between 0.10 and 0.25 times the SD of the time series). The results of Dataset0 and type I subjects after DTW processing are presented, and threshold *r* was selected as 0.10 times the SD of the data. Table 5 summarizes the classifier performance metrics of the two datasets at three settings of cut-point *c* values: optimal cut-point c* (equally weighs sensitivity and specificity), cut-point *c* for highly weighting sensitivity (*Se* > 99%) and highly weighting specificity (*Sp* > 99%). For optimal cut-point c* that equally weighs sensitivity and specificity, indexes *J*, *Se*, *Sp* and *Acc* are 37.13%, 60.57%, 76.56% and 70.95% respectively for traditional SampEn using Dataset0. As for type I after DTW, the same indexes are 75.18%, 90.06%, 85.12% and 88.85% respectively, outperforming Dataset0 thoroughly. For highly weighting sensitivity (*Se* > 99%), we can see that *J*, *Sp* and *Acc* values of type I after DTW are 47.49%, 48.48% and 86.61% respectively, which are better than those from Dataset0. For highly weighting specificity (*Sp* > 99%), the results of traditional SampEn using type I subjects after DTW still present better performance in all indexes except *Acc*. Besides, distribution ranges of SampEn between NSR and CHF groups and ROC plots using the same datasets are also shown in Figure 14. In subplot (A), the overlapping part between NSR and CHF groups in Dataset0 has entirely disappeared in type I after DTW. In subplot (B), the AUC value for type I subjects after DTW is 94.48%, which is over 20% higher than that of Dataset0. Thus, our data refinement processing is also adaptive to SampEn with traditional threshold.

## 5. Discussion

In the past decades, much research focused on the diagnosis as well as evaluation of severity of heart failure using various HRV measures. The evaluation indicators, such as accuracy, in heart failure detection have reached a satisfying level for clinical diagnosis by applying machine learning techniques [47]. Meanwhile, estimating the severity of the heart failure according to commonly employed classifications such as NYHA could also achieve good results with the combination of HRV indices and machine learning algorithms [48]. Nevertheless, when taking the advantage of long-term ECG signals from open access database, little research would refine the data processing and pick out those representative segments. It would also be unusual to divide test subjects into several types without reference to general classification standards. Actually, filtering out redundant segments in 24-h time series would alleviate the processing cost and specify individual characteristics. The consequent classification toward both NSR and CHF subjects would further evaluate the quality of their time series and select subjects with intensive features corresponding to their physiological conditions. This is why we proposed a rough selection plus a fine clustering on the widely used RR interval dataset. The experimental results of applying SampEn and performance metrics have verified that such refinement process would lead to better detection of heart failure.

One issue that requires further discussion is the selection of boundary value for subject extraction. In Section 4.2, we chose the boundary as 90 segments, which was largely based on the analysis of the overall data. With regard to the segments remained after fast HR sequence selection and DTW processing, this boundary could guarantee that there are enough subjects in type I for later classification, and the results would be preferable in this case. Nevertheless, we have to admit that the boundary value has great influence on the classification results, and optimization upon this parameter would be needed once databases with more individuals are applied. Another issue arises concerning the interrelationship between DTW processing and subject extraction. As a delicate refinement, DTW calculation can block out dissimilar segments within each individual, thus enhancing the classification results. However, for any further improvement, it would be powerless to process the whole database with all subjects included. Dividing the subjects into two types and picking out those high-quality ones seem reasonable under the circumstances. Subject extraction has improved the classification results in essence. In that sense, DTW calculation is a fine screening, which could make the already-improved results even better. Therefore, implementing both DTW processing and subject extraction would be advisable.

The poor performance of type II subjects in Section 4.2 is also worth exploring. One possible reason for that might be most segments of a type II subject, no matter healthy or not, present features slightly inclining towards NSR, but not as typical as those NSR in type I. When referring to classification, these segments are more likely to be labeled as NSR by our classifier, which means in most cases only healthy subjects are classified correctly, thus the overall accuracy is no greater than 70%. This also explains why type II subjects have achieved high specificity, while the sensitivity is rather low. Since most segments of type II subjects show unified features, DTW processing could only screen out the minority, thus it is almost ineffective on these subjects. Furthermore, as the proportion of CHF segments in type II subjects is relatively small, the analysis on the characteristics of these time series might not be comprehensive, which also leads to low sensitivity in case of equally-weighted sensitivity and specificity.

Moreover, after the data refinement process, some subjects had fewer RR segments or even no segment remained, thus they were excluded in the analysis later on. This provokes another issue that requires consideration. When we could not extract any RR segment from one individual in clinical applications, leaving out subjects like what we did in a relatively huge database is unreasonable. Thus, it would be advisable to keep measuring for another time period until valuable ECG signals have been captured. Furthermore, we also need to update the selection method in adaption to all kinds of heart failure time series in the future. Since the heart beat dynamics in 24 h varies among individuals [49], we might relax the standard for fast HR sequence selection, and adjust filter indices in accordance with personal heart rate level. Exploring methods to analyze the acceleration and deceleration capacity of the heart in the time series would also be a plausible solution to further research.

There are several limitations in this study. First, only data from NSR RR Interval Database and CHF RR Interval Database were used to examine our proposed method, and the RR segments remained after selection are approximately 14% of the original dataset. Thus, in future work, multiple databases will be used to demonstrate the efficacy of the proposed for heart failure detection. Second, the reasons for increasing heart rate are very complicated. Heart rate could be elevated during exercises or voluntary intentions [50], and the relative fast base heart rate of some people might lead to misclassification in segment selection. In addition, although CHF subjects have cardiac dysfunction, their deceleration and acceleration capacity towards heart rate can be improved through repeated physical training [51]. It seems that our assumption has oversimplified the heart rate acceleration situations. Third, since some heart failure subjects are in the asymptomatic stages [52], their times series data might be very similar to those of healthy people. To dig out the discrepancy between such patients and healthy individuals, the effects of automatic nerve system in the long-term ECG signals have to be explored. Moreover, more parameter combinations, such as larger embedding dimension *m*, various physical threshold *r* and different segment length *N*, should be investigated in the study, to verify the consistency of the proposed method.

In conclusion, the current study proposes a signal refining process based on the 24-h RR interval time series of NSR and CHF subjects. The significance of the proposed method is shown in the evaluation metrics in comparison with the original dataset. By using this method, data analysis volume would reduce considerably as only valid segments are preserved, and the signal characteristics of individuals’ heart condition are clarified after extraction which lead to better anomaly detection. Therefore, the proposed signal refining process would offer a new way to enhance the accuracy of heart failure detection.

## Figures and Tables

**Table 1:** Statistical results of the numbers of RR interval recordings, RR intervals and RR segments from the 54 NSR and 29 CHF RR Interval Databases for original dataset, dataset with fast HR sequence selection, dataset after DTW calculation as well as subject extraction.

Variables	NSR Group	CHF Group
No. of RR interval recordings	54	29
No. of RR intervals	5,790,504	3,312,195
No. of RR intervals after removing greater than 2 s	5,780,148	3,306,394
No. of RR intervals after removing abnormal heartbeats	5,738,937	3,102,120
No. of RR segments (*N* = 300) after removing abnormal heartbeats	19,101	10,324
No. of RR intervals after fast HR sequence selection	632,100	775,200
No. of RR segments (*N* = 300) after fast HR sequence selection	2107	2584
No. of RR segments (*N* = 300) after DTW calculation	1718	2411
No. of RR segments (*N* = 300) for type I subjects	625	1921
No. of RR segments (*N* = 300) for type II subjects	1093	490

**Table 2:** Results of SampEn from the parameter combination of embedding dimension *m* = 1 and 2, physical threshold *r* = 12 ms, and segment length *N* = 300. Herein, three datasets are used: Dataset0 with all RR segments preserved, Dataset1 with only fast HR sequences preserved, and Dataset2 with DTW processing added. *P*-value measured the statistical significance between the NSR and CHF groups at each combination of (*m*, *r*). Data are expressed as number or mean ± standard deviation (SD). ‘*’: statistical significance *P* < 0.01.

	SampEn of Dataset0			SampEn of Dataset1			SampEn of Dataset2		
	NSR	CHF	*p*-Value	NSR	CHF	*p*-Value	NSR	CHF	*p*-Value
*m = 1*	1.06 ± 0.22	0.72 ± 0.28	7 × 10^−8^ *	0.70 ± 0.13	0.46 ± 0.11	8 × 10^−321^ *	0.72 ± 0.12	0.46 ± 0.10	2 × 10^−319^ *
*m = 2*	0.97 ± 0.21	0.63 ± 0.28	2 × 10^−8^ *	0.59 ± 0.12	0.38 ± 0.09	3 × 10^−297^ *	0.61 ± 0.11	0.38 ± 0.09	8 × 10^−302^ *

**Table 3:** Sensitivity, specificity and accuracy results of SampEn using Dataset0, Dataset1 and Dataset2 respectively. In the parameter combination, embedding dimension *m* was set as 1, physical threshold *r* = 12 ms, and segment length *N* = 300.

Metric	Equally-Weighted *Se* and *Sp*			Highly-Weighted *Se*			Highly-Weighted *Sp*		
	Dataset0	Dataset1	Dataset2	Dataset0	Dataset1	Dataset2	Dataset0	Dataset1	Dataset2
*c*	0.72	0.57	0.59	1.86	0.93	0.92	0.40	0.26	0.26
*J(%)*	43.35	49.14	53.83	1.80	9.71	11.17	16.86	12.36	12.59
*Se(%)*	60.31	75.39	79.10	>99.0	>99.0	>99.0	17.85	13.31	13.52
*Sp(%)*	83.05	73.75	74.74	2.80	10.68	12.17	>99.0	>99.0	>99.0
*Acc(%)*	75.07	74.65	77.28	36.55	59.35	62.87	70.53	51.82	49.12

**Table 4:** Sensitivity, specificity and accuracy results of SampEn before and after DTW processing. Subjects with RR segment number ≥90 (type I) and RR segment number <90 (type IIII) are set as contrast here. In the parameter combination, embedding dimension *m* was set as 1, physical threshold *r* = 12 ms, and segment length *N* = 300.

Metric	Equally-Weighted *Se* and *Sp*		Highly-Weighted *Se*		Highly-Weighted *Sp*	
	Before DTW	After DTW	Before DTW	After DTW	Before DTW	After DTW
***type I***						
*c*	0.59	0.61	0.74	0.74	0.35	0.48
*J(%)*	66.49	78.93	46.66	58.05	32.00	60.93
*Se(%)*	88.26	91.41	>99.0	>99.0	32.96	61.89
*Sp(%)*	78.24	87.52	47.65	59.04	>99.0	>99.0
*Acc(%)*	84.85	90.46	81.56	89.20	55.41	71.01
***type II***						
*c*	0.41	0.41	1.16	1.18	0.24	0.24
*J(%)*	10.24	12.72	3.05	2.84	2.30	2.35
*Se(%)*	20.86	21.22	>99.0	>99.0	3.24	3.27
*Sp(%)*	89.38	91.49	3.95	3.66	>99.0	>99.0
*Acc(%)*	65.86	69.74	36.60	33.23	66.17	69.43

**Table 5:** Sensitivity, specificity and accuracy results of SampEn with traditional threshold before and after data refinement processing. Dataset0 refers to original RR interval databases, and type I after DTW refers to subjects with RR segment number ≥90 after both fast HR sequence selection and DTW calculation. In the parameter combination, embedding dimension *m* was set as 1, traditional threshold *r* = 0.10, and segment length *N* = 300.

Metric	Equally-Weighted *Se* and *Sp*		Highly-Weighted *Se*		Highly-Weighted *Sp*	
	Dataset0	Type I after DTW	Dataset0	Type I after DTW	Dataset0	Type I after DTW
*c*	0.68	1.57	2.76	1.75	0.89	1.35
*J(%)*	37.13	75.18	1.74	47.49	0.24	50.21
*Se(%)*	60.57	90.06	>99.0	>99.0	1.24	51.17
*Sp(%)*	76.56	85.12	2.74	44.48	>99.0	>99.0
*Acc(%)*	70.95	88.85	36.51	86.61	64.70	62.92
